# The WHO-TDR Dissemination and Implementation Massive Open Online Course (MOOC): Evaluation and Lessons Learned from Eight Low-and Middle-Income Countries

**DOI:** 10.21203/rs.3.rs-1455034/v1

**Published:** 2022-03-29

**Authors:** Ashlin Rakhra, Cole Hooley, Meredith Fort, Mary Beth Weber, LeShawndra Price, Hoa L. Nguyen, Manuel Ramirez, Adamson S. Muula, Mina Hosseinipour, Kingsley Apusiga, Victor Davila-Roman, Joyce Gyamfi, Kezia Gladys Amaning Adjei, Josephine Andesia, Annette Fitzpatrick, Pascal Launois, Ana A. Baumann

**Affiliations:** New York University Grossman School of Medicine; Brigham Young University; Colorado School of Public Health American Indian and Alaska Native Programs: Colorado School of Public Health Centers for American Indian and Alaska Native Health; Rollins School of Public Health: Emory University School of Public Health; National Institute of Allergy and Infectious Diseases; University of Massachusetts Chan Medical School; Institute of Nutrition of Central America and Panama: Instituto de Nutricion de Centroamerica y Panama; University of Malawi College of Medicine; University of North Carolina at Chapel Hill School of Medicine; Kwame Nkrumah University of Science and Technology; Washington University in St Louis School of Medicine; New York University College of Global Public Health; Kwame Nkrumah University of Science and Technology; Moi University; University of Washington School of Public Health; Special Programme for Research and Training in Tropical Diseases; Washington University in St Louis Department of Surgery

**Keywords:** massive open online course (MOOC), dissemination and implementation, capacity building, low-and middle-income countries

## Abstract

**Introduction:**

Non-communicable diseases (NCDs) are a leading cause of morbidity and mortality in low-and middle-income countries (LMICs). Despite this, a lack of funding, training and mentorship for NCD investigators in LMICs exists. In an effort to gain knowledge and skills to address these gaps, participants from the Global Research on Implementation and Translation Science (GRIT), a consortium of studies in eight LMICs and their networks, attended the dissemination and implementation (D&I) massive open online course (MOOC) developed by the Special Programme for Research and Training in Tropical Diseases at the World Health Organization to strengthen D&I capacity building. Here, we report on the feasibility of this MOOC, which was implemented during the SARS COVID-19 pandemic from April- November 2020.

**Methods:**

Participants completed pre- and post- training questionnaires to assess self-reported D&I competencies, general research skills, and research mentor access and quality. D&I competencies were measured by use of a scale developed for a US-based training program, with change in competency scores assessed by paired t test. We used univariate statistics to analyze the data for all other outcomes.

**Results:**

Of the 247 participants enrolled, 32 (13%) completed all MOOC components. D&I competency scores suggest improvement for those who had complete pre- and post-assessments. Trainee’s average score on the full competency scale improved 1.45 points (0–5 scale) from pre- to post-test; all four subscales also showed evidence of improvements. There were small but not significant increases in competencies for grant writing, proposal/ manuscript writing and presentations from pre- to post-test assessment. 40% of trainees reported access to a research mentor and 12% reported access to a D&I specific mentor. Participants reported barriers (e.g., unstable internet access and challenges due to COVID-19) and facilitators (e.g., topical interests, collaboration with colleagues) to completing the MOOC.

**Conclusions:**

Although COVID-19 affected program usage and completion, the MOOC was feasible and effective, showing that among LMIC participants completing the course, there was improvement in D&I competency scores. Recommendations for future D&I trainings in LMICs should include 1) adding more topic specific modules (i.e., NCD research, general research skills) for scalability; 2) fostering more collaboration with participants across LMICs; and 3) establishing partnerships with D&I mentors for course participants.

## Introduction

Non-communicable diseases (NCDs) are the leading cause of mortality worldwide that disproportionately impact low and middle-income countries (LMICs).^(1)^With 80% of deaths from NCDs occurring in LMICs, the role of local research capacity and relevant research informing policy and practice is crucial.^(2)^Despite this, there has been a particular lack of funding, training and mentorship for NCD investigators in LMICs.^(3)^

The Special Programme for Research and Training in Tropical Diseases (TDR) at the World Health Organization (WHO) developed the massive open online course (MOOC), which aims to disseminate implementation research concepts.^(4)^The primary goal of the course is to strengthen capacity building and improve training opportunities, targeting local public health researchers, practitioners and policymakers.^(4)^The course delivers implementation research education in LMICs where access to formal learning pathways, such as university courses in implementation research, may be limited.^(5)^Investing in research capacity and training in LMICs reduces disease burden by building local research capacity and ensuring that those who are being trained are best equipped to address the needs of their communities.^(6–9)^

MOOCs have steadily gained popularity given the accessibility, affordability, and effectiveness of the courses.^(4, 10, 11)^The TDR MOOC on Implementation Research improved participant knowledge and understanding of implementation research and increased participants’ ability to apply the course concepts to professional practice.^(12)^While this MOOC was developed with a focus on infectious diseases of poverty, the course concepts can be applied to strengthening implementation research capacity for NCDs and other disciplines.^(12, 13)^

The goals of this paper are to describe the evaluation outcomes of one of the 2020 MOOC-D&I trainings conducted by the Global Research on Implementation and Translation Science (GRIT) consortium as part of the GRIT’s ongoing mentorship and capacity building programs, and share barriers, facilitators, and recommendations to enhance future D&I training opportunities in LMICs.

## Methods

### TDR MOOC on IR

The TDR MOOC on IR is a step-by-step online training for public health researchers and decision-makers that focuses on design and implementation of research projects.^(12)^ Core concepts of implementation research are addressed in five modules including: 1) identifying the challenges of various health settings; 2) assessing the appropriateness of existing disease control strategies; 3) developing new interventions and strategies by working with communities and stakeholders; 4) specifying implementation research questions; and 5) designing rigorous research projects, including how to identify implementation outcomes, evaluating effectiveness, and making plans to scale-up implementation in real life settings.^(12)^ The course includes homework assignments, the requirement of completing and passing at least four quizzes and a final assignment with a peer-review component. Participants completed an electronic survey at the beginning and conclusion of the MOOC to evaluate the change in knowledge and self-assessed competencies. Subsequently, participants were asked to share barriers and facilitators to completing the MOOC.

### Participants

There were two sets of participants in this study. The first were participants from the Global Research on Implementation and Translation Science (GRIT) Consortium funded by the National Heart Lung and Blood Institute (NHLBI). The consortium consists of research teams from eight countries, five of which (Guatemala, Ghana, Kenya, India, and Vietnam) test implementation strategies to deliver evidence-based interventions within these countries for the prevention, treatment, and control of hypertension (HyTREC sites) and three of which (Malawi, Nepal, and Rwanda) provide capacity building in NCD and D&I research needed to close the gap between research and practice (TREIN sites).^(3, 6)^ Specifically, all countries have partnership between D&I mentors and hypertension physicians in the U.S. and in country. The stakeholders from all countries are invited to GRIT workshops about implementation science and hypertension care, and all countries have developed formal and informal infrastructures of mentoring in D&I and research in general.^(14)^ The MOOC was an added structure in which consortium members decided to engage to support enhancing D&I knowledge for GRIT members.

The invitation to participate in the MOOC was open to all consortium members. Additionally, GRIT participants were encouraged to share the announcement with their respective networks. The second set of participants were not part of the GRIT Consortium and were recruited through snowball sampling through the GRIT network. We did not have inclusion or exclusion criteria. Our recruitment email invited anyone interested in the MOOC with a brief description of the course, timeline and expectations. Enrollment was open from April 6 to May 5, 2020 and the course ran from May 11, 2020 to November 6, 2020.

### Measures

The primary outcome of this study was competency in D&I research. Surveys were distributed via Qualtrics.^(15)^ We also examined four secondary outcomes including: (1) D&I mentor access and quality; (2) general research mentor access and quality; (3) general research skill competencies, in manuscript writing, proposal writing, making scholarly presentations, and grant writing; and (4) a qualitative assessment of barriers and facilitators to completing the MOOC. While the TDR MOOC does not have a formal mentorship as part of the course, GRIT members are connected formally or informally with their D&I stakeholders in either delivering interventions (HyTREC sites) or enhancing capacity building in D&I and HTN care (TREIN sites). Additionally, unique to this training was the expectation that results from the MOOC training could be used as potential future research ideas as part of GRIT capacity building efforts.

The current study is a single-group, pre-post study design to assess changes in D&I research competencies, measure mentor access and quality, and describe general research skills among participants in the TDR MOOC. Additionally, barriers and facilitators to completing the course were examined. Researchers originally developed the competency measure for D&I trainings in the United States.^(5, 8, 16)^ Others have subsequently used this measure to assess D&I competencies for the WHO MOOC internationally.^(10, 17)^ The 43 item self-report measure is organized into four subscales: (1) definitions, background, and rationale, (2) theory and approaches, (3) design and analysis, and (4) practice-based considerations^(5, 8)^ using a 5-point Likert scale (i.e., Not at all to Extremely).

A secondary outcome of this study was D&I mentor access and quality, measured through three questions added to the original survey. The first question asked trainees whether they had access to a D&I mentor (answer options: yes, no, not sure). If the trainee had a D&I mentor, they were asked two follow-up questions. The first follow-up question assessed the quality of the mentoring (“how would you rate the overall quality of the mentoring you received from your D&I mentor?”). Trainees answered using a 7-point Likert scale with anchoring verbiage at 3 points (1-very low, 7-very high). The second follow-up question assessed the degree to which the D&I mentoring met their expectations: “to what extent do you feel your D&I mentor is meeting your expectations?” Trainees answered using a 7-point Likert scale with anchoring verbiage at 3 points (1-Not at all, 7-Completely).

A third outcome examined the general research mentor access and quality, with three additional survey questions. The first research mentor question asked trainees whether they had access to a research mentor (answer options: yes, no, not sure). Trainees with a research mentor were asked a follow-up question about mentoring quality and the degree to which the mentoring met their expectations. The same questions, with answer options, that were asked to assess the quality of the mentorship and met expectations for their general research mentor were asked for those with a D&I specific mentor.

A fourth outcome measured general research skill competencies, in manuscript writing, proposal writing, making scholarly presentations, and grant writing. Trainees rated their level of competency for each of these items using a 5-point Likert scale (1 not at all to 5 extremely).

The final outcome examined was barriers and facilitators to completing the MOOC. Trainees who completed the MOOC were asked: “what enabled you to complete the MOOC?” Trainees who completed some but not all of the MOOC were asked: “what enabled you to complete some of but not all the MOOC components?” Trainees who did not complete all of the MOOC were asked: “what prevented you from being able to complete the MOOC?” All trainees were asked: “What changes/support would help future participants complete the MOOC?” The questions pertaining to barriers and facilitators were open-ended and included in the post-assessment survey.

MOOC participant demographic information was also collected. Specifically, participants provided their gender, age, education, country, work position, work location, and GRIT participation.

### Analysis

Quantitative analyses were conducted in Stata 16.1. A paired t-test was used to determine if trainees’ D&I competencies and general research competencies changed from pre- to post-test. The trainees’ average total D&I competency score and their average scores for each sub-scale were calculated.^(8, 10)^ The analytic sample only included trainees with complete pre- and post- D&I competency measures; those with missing data were excluded. Chi-square tests, Fisher exact tests, and independent two sample t-tests using demographic variables were obtained to determine if the trainees without complete D&I competency measures differed from those with complete pre- and post-measures. Some respondents had missing demographic variables and could not be included in the comparison assessment. Demographic variable tests were run separately; the lowest number of missing variables was 5 and the highest was 9. The results from that analysis suggest that there were no meaningful differences between those with complete D&I competency scores and those without. As such, only the results for trainees with complete pre- and post-test D&I competency measures are reported. The same tests were run on the general research competency completers and non-completers. No meaningful difference was found between the two groups. As such, only the respondents with complete pre/post general competency responses were included for analysis of the fourth outcome.

A univariate statistic was used to analyze the data for the second and third outcomes. Data for those outcomes came from the pre-test survey data. Observations with missing data were removed. Finally, the qualitative data for the final outcome, barriers and facilitators, were analyzed using thematic analysis to identify patterns and areas of overlap in participant responses.^(18)^

## Results

### MOOC Participation

**Figure 1:**
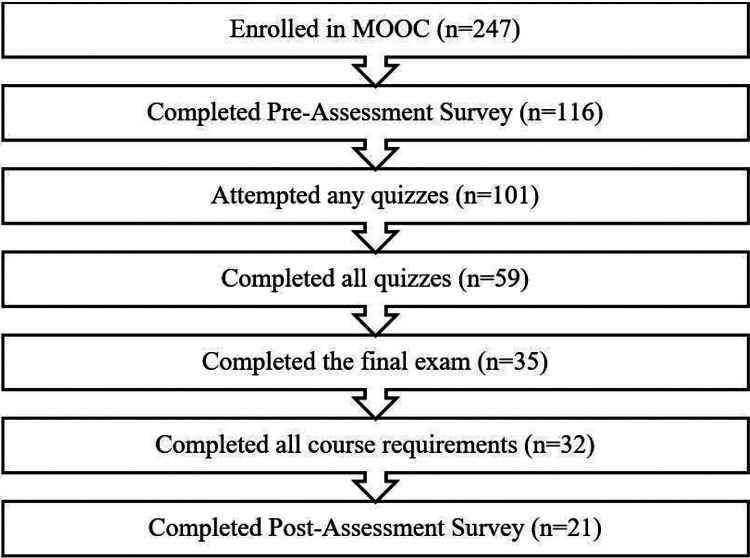
Participant Retention

As outlined in Fig. 1, 247 individuals from the GRIT Consortium and ancillary networks enrolled in the MOOC; 116 completed the pre-assessment survey, 101 attempted any quizzes, 59 completed all quizzes, 35 completed the final exam, and 32 participants completed all course requirements.

Table 1 outlines the demographic characteristics of the trainees who completed both the pre- and post-competency measures (*n* = 21) and the demographics for all participants that initially enrolled in the MOOC (*n* = 116). Most of the trainees with complete pre-and post-D&I competency measures were female (57%); had a master’s degrees (76%); were from Rwanda (33%) and Malawi (24%); were not GRIT Consortium members (62%); did not have previous D&I training experience (67%); and the average participant age was 35 ± 4.5.

### D&I Competencies

D&I competency scores showed evidence of improvement for those with complete pre- and post-competency scores (see Table 2). Trainees’ average score on the full scale improved 1.45 points from pre-to post-test (2.12 ± 0.93 vs 3.57 ± 0.97, *t*(20) = 7.42, *p* < 0.001). Scores on the first subscale, *definitions, background, and rationale*, improved by 1.36 points (2.54 ± 0.93 vs 3.90 ± 0.94, *t*(20) = 6.60, *p* < 0.001). Scores on the second subscale, *theory and approaches*, improved by 1.63 points (2.01 ± 0.98 vs 3.64 ± 1.06, *t*(20) = 6.23, *p* < 0.001). Scores on the third subscale, *design and analysis*, improved by 1.45 points (1.94 ± 0.95 vs 3.39 ± 1.02, *t*(20) = 7.94, *p* < 0.001). Scores on the final subscale, *practice-based considerations*, improved by 1.37 points (2.08 ± 1.02 vs 3.45 ± 0.94, *t*(20) = 7.05, *p* < 0.001).

Participants reported that access to D&I mentoring was low (not reported in Table). Only 12% (n = 14) of the MOOC trainees who completed the pre-survey (n = 111) indicated that they had a D&I mentor. Of those that had a D&I mentor, 63% reported the quality of mentoring was above average, with 21% rating the quality as “very high.” 71% reported a 4 or 5 out of 7 regarding that their mentor met their expectations for mentorship, where 4 reflected moderately meeting expectations.

Access to general research mentoring was reported by the participants as being higher compared to D&I mentoring. 40% (n = 46) of the MOOC trainees that completed the pre-survey reported having a research mentor. Around 52% of those with a research mentor rated the mentoring quality as above average. Similar to the D&I mentoring, 46% reported a 4 or 5 out of 7 reflecting that the mentor met expectations for mentorship, where 4 represented moderately meeting expectations.

Research competency scores among those with complete pre- and post-survey responses (n = 33) varied. While the scores generally improved from pre- to post-survey, the differences were not statistically meaningful. Participant manuscript writing scores remained constant between pre- and post-test (3.5 vs. 3.5). Proposal writing improved from 3.4 to 3.5. Scholarly presentation scores improved from 3.6 to 3.8. Grant writing scores improved from 2.2 to 2.4.

### Barriers and Facilitators

Table 3 outlines the barriers and facilitators identified by participants in the post-assessment. Participants reported major barriers preventing them from completing the course including lack of time, other work commitments or additional responsibilities placed on them due to the COVID-19 pandemic, and lack of stable and consistent internet connection. Participants identified time management skills, an interest in the topics addressed by the course, and recognizing the opportunity to learn as driving factors in completing the course. Additional facilitators included collaborating with other participants, supervisors and colleagues; the course flexibility (i.e., pre-recorded sessions to adapt to participant’s schedule as opposed to live sessions); and increased time to work on the course due to personal or professional changes during the COVID-19 pandemic.

### Course Recommendations

Participant recommendations for future MOOC sessions included: 1) greater mentorship from the GRIT stakeholders throughout the course; 2) greater collaboration among participants across LMICs; 3) having the ability to retake modules or quizzes for greater understanding of a specific topic; 4) incorporating an NCD module or more NCD related examples; 5) minimizing website navigation challenges; 6) facilitating access to a reliable internet connection; and 7) more course flexibility. To enhance flexibility, participants suggested having a flexible deadline for the peer assessment, having all modules accessible at the beginning of the course with a final deadline, and having extra time for assignments.

## Discussion

This study examined the experience of participants from eight LMICs in one of the 2020 TDR MOOC on IR. Using the data from the pre-and post-assessment surveys, the self-reported D&I competencies were analyzed as well as barriers and facilitators to completing the course, which provide recommendations and implications for future MOOCs. Although there was a low retention rate in the MOOC, participants completing the post survey showed improvements in their D&I competencies. Participants reported low access to D&I mentors, limited access to general research mentors, and low self-reported competency for manuscript and scientific writing.

The course completion rate was likely impacted by a couple of variables. First, internet access was a major barrier for retention in this study, which has been shown in similar studies.^(10)^ MOOCs, by design, enroll large groups of students, including both active and passive participants. Reconceptualizing retention to only include participants who substantively engaged with the course ^(19)^ might provide a more accurate picture of program metrics. Third, the timeline in which we started the MOOC was challenging. Enrollment took place in April 2020 with a course start date in May 2020, right before several of the participating countries started the lock down to prevent further spread of the SARS-CoV-2 virus. Fourth, as soon as the cohort started the training, the MOOC website moved to be hosted by another company and the transition posed some issues with access to the videos. With the larger movement towards online courses and trainings, future guardrails to develop and maintain websites for online learning will be important.^(20–22)^

Due to the global uncertainty of the COVID-19 pandemic coupled with TDR platform issues, the first module was extended three months until the end of July 2020. The remaining modules adhered to the original timelines, with a spacing of two weeks between each module. During this period, 53% of enrolled participants no longer engaged in course activities. When asked about the barriers for participation in the post-assessment survey, participants shared that the lack of stable internet, other work commitments and responsibilities, and needing more time to complete the course were key barriers that affected their participation in this course. These barriers have been reported by participants from previous MOOCs,^(10, 13, 17)^ but they were likely intensified by the COVID-19 pandemic and associated lockdowns in this past year.

The results indicate strong evidence of improvement with self-reported D&I competencies similar to previous courses.^(10)^ The subscale that had the largest change was *theory and approaches* and the subscale with the least change was *definitions, background and rationale* and *practice-based considerations*. These results differ from other D&I trainings where a majority of participants reported the largest change in the *definitions, background, and rationale* subscale.^(8, 10)^ The difference in results may be related to the composition of participants, where 33% of participants in this study had previous D&I training before the course. The general research competency scores in manuscript writing, proposal writing, and giving scholarly presentations did not change in a meaningful way from pre-to-post test. These findings suggest that general research capacities, not specific to D&I, should be targeted by future capacity-building activities, particularly grant writing. Accordingly, the TDR WHO has developed a flexible and interactive D&I toolkit to support capacity building and proposal writing.^(23)^

The need for increased mentoring and guidance was a prominent theme in the recommendations submitted by the participants, as only 14% reported having a mentor in D&I research. Even though every country has a D&I consultant, the limited access to D&I mentors may be a reflection of very few researchers being trained in the emerging field of implementation science in LMICs. Evaluation of other D&I trainings in high resource settings have shown the importance of networking and mentoring, as well as time, for the development of academic outcomes,^(8, 24)^ and previous MOOCs with added support beyond mentorship (i.e., meetings for participants to discuss modules, Q&A sessions, discussion forums) demonstrated an increase in participant engagement.^(10, 25)^ Future D&I capacity trainings in LMICs should include greater mentorship and support throughout the course as it could contribute to higher course completion and improved overall D&I competency reporting.^(7, 26)^

### Limitations

The major limitation of this paper is the small number of participants that completed the course. Additionally, evaluation data was comprised of only self-report data and, therefore subjected to bias and social desirability. Lastly, we did not follow up with participants to ask whether they were able to apply what they learned. The lack of opportunity to practice what they learned has been a challenge described by participants in previous MOOCs.^(24, 27)^

### Future Directions and Implications

Despite the challenges and limitations, partnering with the Special Programme for Research and Training in TDR MOOC is a feasible and scalable strategy to increase D&I training in LMICs. The use of D&I competency metrics allows for further evaluation on how to design training in D&I. In the future, research partners may add specific modules, such as HTN care and D&I grant writing. Some of this is already being done as part of capacity building initiatives.^(17, 28)^ In moving forward, setting up and strengthening a collaborative practice whereby mentoring and peer collaboration across countries could be beneficial to all in enhancing the capacity for D&I research training.^(28)^

## Conclusions

This study describes the 2020 massive open online course (MOOC) for dissemination and implementation (D&I) developed by the TDR. This study contributes to the growing literature in MOOCs being effective training opportunities reflected in the increase of self-reported D&I competencies from participants.

## Supplementary Material

Supplement 1

## Figures and Tables

**Table 1:** Demographic characteristics of trainees with complete pre- and post-test D&I competency scores (n = 21), and individuals who answered at least one question in the pre-test (n = 116).

	With complete pre/post D&I competency (*n* = 21)	At least one item in pre-test survey (*n* = 116)
**Gender, n(%)**		
Male	9 (43%)	61 (53%)
Female	12 (57%)	49 (42%)
Other	0	1 (1%)
Missing	0	5 (4%)
**Education, n(%)**		
PhD/MD	2 (10%)	18 (16%)
Master’s degree	16 (76%)	61 (53%)
Some graduate school	0	4 (3%)
Bachelor’s degree	3 (14%)	27 (23%)
Some college	0	1 (1%)
Missing	0	5 (4%)
**Country, n(%)**		
Ghana	3 (14%)	9 (8%)
Guatemala	0	4 (3%)
India	0	4 (3%)
Kenya	0	10 (9%)
Malawi	5 (24%)	22 (19%)
Nepal	4 (19%)	10 (9%)
Rwanda	7 (33%)	45 (39%)
Vietnam	2 (10%)	7 (6%)
Missing	0	5 (4%)
**Work position, n(%)**		
Academic	7 (33%)	36 (31%)
Clinician	2 (10%)	10 (9%)
Leadership	0 (0%)	17 (15%)
Research (other)	6 (29%)	27 (23%)
Multiple positions	3 (14%)	9 (8%)
Other	2 (10%)	8 (7%)
Missing	1 (5%)	9 (8%)
**Work location, n(%)** ^a^		
Ministry of health	3 (14%)	14 (12%)
Research center	5 (24%)	25 (22%)
University	12 (57%)	54 (47%)
WHO	1 (5%)	1 (1%)
Community health center	0 (0%)	3 (3%)
Hospital	3 (14%)	28 (24%)
Other	0 (0%)	8 (7%)
Missing	1 (5%)	9 (8%)
**GRIT participants, n(%)**		
TREIN	6 (29%)	16 (14%)
HyTREC	2 (10%)	19 (16%)
Not part of GRIT	13 (62%)	74 (64%)
Missing	0	7 (6%)
**Previous D&I training, n(%)**		
Yes	7 (33%)	38 (33%)
No	14 (67%)	73 (63%)
Missing	0	5 (4%)
**Age, m ± sd (range)**	35 ± 4.5 (25–42)	35 ± 5.8 (25–63)^b^

a = values sum to more than 100% because respondents could select multiple work locations

b = sample size for the age was (n = 111)

**Table 2:** D&I Competencies, pre- to post-test change in average scores (1 = not at all, 5 = extremely, n = 21)

D&I Research Competency Areas	Pre-test (Mean ± SD)	Post-test (Mean ± SD)	95% CI mean difference
Full scale	2.12 ± .93	3.57 ± .97	1.04–1.86*
Definitions, background, and rationale	2.54 ± .93	3.90 ± .94	.93–1.79*
Theory and approaches	2.01 ± .98	3.64 ± 1.06	1.08–2.17*
Design and analysis	1.94 ± .95	3.39 ± 1.02	1.07–1.83*
Practice-based considerations	2.08 ± 1.02	3.45 ± .94	.97–1.78*

Note:

*= p < .001

**Table 3:** Barriers and Facilitators to Completing the MOOC.

Barriers	Facilitators
Lack of time	More time to work on the course due to COVID
Other commitments (work/ COVID related)	Time management
Lack of internet/ unstable connection	The learning opportunity
Completed previous MOOC with similar content	Interest in the topic
	Reminders by supervisors
	Working with colleagues on the course
	Pre-recorded sessions to watch when time permitted as opposed to live sessions

## Data Availability

Data are available upon request.

## Key Messages Regarding Feasibility

- What uncertainties existed regarding the feasibility? The small number of participants that completed the course and evaluation data comprised of only self-report data and, therefore subjected to bias and social desirability were the main uncertainties. Additionally, we did not follow up with participants to ask whether they were able to apply what they learned. The lack of opportunity to practice what they learned has been a challenge described by participants in previous MOOCs.
- What are the key feasibility findings? Although COVID-19 affected program usage and completion, the MOOC was feasible and effective, showing that among LMIC participants completing the course, there was improvement in D&I competency scores. Trainee’s average score on the full competency scale improved 1.45 points (0–5 scale) from pre- to post-test; all four subscales also showed evidence of improvements. There were small but not significant increases in competencies for grant writing, proposal/ manuscript writing and presentations from pre- to post-test assessment. Participants reported barriers (e.g., unstable internet access and challenges due to COVID-19) and facilitators (e.g., topical interests, collaboration with colleagues) to completing the MOOC.
- What are the implications of the feasibility findings for the design of the main study? Despite the challenges and limitations, partnering with the Special Programme for Research and Training in TDR MOOC is a feasible and scalable strategy to increase D&I training in LMICs. The use of D&I competency metrics allows for further evaluation on how to design training in D&I. This study contributes to the growing literature in MOOCs being effective training opportunities reflected in the increase of self-reported D&I competencies from participants.

## List Of Abbreviations

D&I: Dissemination and Implementation
GRIT: Global Research on Implementation and Translation Science
LMICs: Low and middle-income countries
MOOC: Massive open online course
NCDs: Non communicable diseases
TDR: Training in Tropical Diseases
WHO: World Health Organization
